# Liposome: classification, preparation, and applications

**DOI:** 10.1186/1556-276X-8-102

**Published:** 2013-02-22

**Authors:** Abolfazl Akbarzadeh, Rogaie Rezaei-Sadabady, Soodabeh Davaran, Sang Woo Joo, Nosratollah Zarghami, Younes Hanifehpour, Mohammad Samiei, Mohammad Kouhi, Kazem Nejati-Koshki

**Affiliations:** 1 Department of Medical Nanotechnology, Faculty of Advanced Medical Science, Tabriz University of Medical Sciences, Tabriz 51664, Iran; 2 Department of Biology, Science and Research Branch, Islamic Azad University, Tehran, Iran; 3 Department of Endodontics, Dental School, Tabriz University of Medical Sciences, Tabriz, Iran; 4 Department of Physics, Tabriz Branch, Islamic Azad University, Tabriz, Iran; 5 School of Mechanical Engineering, WCU Nanoresearch Center, Yeungnam University, Gyeongsan 712-749, South Korea

**Keywords:** Liposomes, Glycolipids, Drug formulations, Drug delivery systems

## Abstract

Liposomes, sphere-shaped vesicles consisting of one or more phospholipid bilayers, were first described in the mid-60s. Today, they are a very useful reproduction, reagent, and tool in various scientific disciplines, including mathematics and theoretical physics, biophysics, chemistry, colloid science, biochemistry, and biology. Since then, liposomes have made their way to the market. Among several talented new drug delivery systems, liposomes characterize an advanced technology to deliver active molecules to the site of action, and at present, several formulations are in clinical use. Research on liposome technology has progressed from conventional vesicles to ‘second-generation liposomes’, in which long-circulating liposomes are obtained by modulating the lipid composition, size, and charge of the vesicle. Liposomes with modified surfaces have also been developed using several molecules, such as glycolipids or sialic acid. This paper summarizes exclusively scalable techniques and focuses on strengths, respectively, limitations in respect to industrial applicability and regulatory requirements concerning liposomal drug formulations based on FDA and EMEA documents.

## Review

### Introduction

Liposomes are small artificial vesicles of spherical shape that can be created from cholesterol and natural non-toxic phospholipids. Due to their size and hydrophobic and hydrophilic character(besides biocompatibility), liposomes are promising systems for drug delivery. Liposome properties differ considerably with lipid composition, surface charge, size, and the method of preparation (Table 1). Furthermore, the choice of bilayer components determines the ‘rigidity’ or ‘fluidity’ and the charge of the bilayer. For instance, unsaturated phosphatidylcholine species from natural sources (egg or soybean phosphatidylcholine) give much more permeable and less stable bilayers, whereas the saturated phospholipids with long acyl chains (for example, dipalmitoylphos phatidylcholine) form a rigid, rather impermeable bilayer structure [1-3].

**Table 1:** **Advantages and disadvantages of liposome **[ [19]]

**Advantages of liposome**	**Disadvantages of liposome**
Liposomes increased efficacy and therapeutic index of drug (actinomycin-D)	Low solubility
Liposome increased stability via encapsulation	Short half-life
Liposomes are non-toxic, flexible, biocompatible, completely biodegradable, and non-immunogenic for systemic and non-systemic administrations	Sometimes phospholipid undergoes oxidation and hydrolysis-like reaction
Liposomes reduce the toxicity of the encapsulated agent (amphotericin B, Taxol)	Leakage and fusion of encapsulated drug/molecules
Liposomes help reduce the exposure of sensitive tissues to toxic drugs	Production cost is high
Site avoidance effect	Fewer stables
Flexibility to couple with site-specific ligands to achieve active targeting	

It has been displayed that phospholipids impulsively form closed structures when they are hydrated in aqueous solutions. Such vesicles which have one or more phospholipid bilayer membranes can transport aqueous or lipid drugs, depending on the nature of those drugs. Because lipids are amphipathic (both hydrophobic and hydrophilic) in aqueous media, their thermodynamic phase properties and self assembling characteristics influence entropically focused confiscation of their hydrophobic sections into spherical bilayers. Those layers are referred to as lamellae [4]. Generally, liposomes are definite as spherical vesicles with particle sizes ranging from 30 nm to several micrometers. They consist of one or more lipid bilayers surrounding aqueous units, where the polar head groups are oriented in the pathway of the interior and exterior aqueous phases. On the other hand, self-aggregation of polar lipids is not limited to conventional bilayer structures which rely on molecular shape, temperature, and environmental and preparation conditions but may self-assemble into various types of colloidal particles [5].

Liposomes are extensively used as carriers for numerous molecules in cosmetic and pharmaceutical industries. Additionally, food and farming industries have extensively studied the use of liposome encapsulation to grow delivery systems that can entrap unstable compounds (for example, antimicrobials, antioxidants, flavors and bioactive elements) and shield their functionality. Liposomes can trap both hydrophobic and hydrophilic compounds, avoid decomposition of the entrapped combinations, and release the entrapped at designated targets [6-8].

Because of their biocompatibility, biodegradability, low toxicity, and aptitude to trap both hydrophilic and lipophilic drugs [9] and simplify site-specific drug delivery to tumor tissues [10], liposomes have increased rate both as an investigational system and commercially as a drug-delivery system. Many studies have been conducted on liposomes with the goal of decreasing drug toxicity and/or targeting specific cells [11-13].

Liposomal encapsulation technology (LET) is the newest delivery technique used by medical investigators to transmit drugs that act as curative promoters to the assured body organs. This form of delivery system proposal targeted the delivery of vital combinations to the body. LET is a method of generating sub-microscopic foams called liposomes, which encapsulate numerous materials. These ‘liposomes’ form a barrier around their contents, which is resistant to enzymes in the mouth and stomach, alkaline solutions, digestive juices, bile salts, and intestinal flora that are generated in the human body, as well as free radicals. The contents of the liposomes are, therefore, protected from oxidation and degradation. This protective phospholipid shield or barrier remains undamaged until the contents of the liposome are delivered to the exact target gland, organ, or system where the contents will be utilized [14].

Clinical medication keeps an enormously broad range of drug molecules at this time in use, and new drugs are added to the list every year. One of the main aims of any cure employing drug is to increase the therapeutic index of the drug while minimizing its side effects. The clinical usefulness of most conservative chemotherapeutics is restricted either by the incapability to deliver therapeutic drug concentrations to the target soft tissue or by Spartan and harmful toxic side effects on normal organs and tissues. Different approaches have been made to overcome these difficulties by providing the ‘selective’ delivery to the target area; the ideal solution would be to target the drug alone to those cells, tissues, organs that are affected by the disease. Selected carriers, for instance colloidal particulates and molecular conjugates, can be appropriate for this determination. Colloidal particulates result from the physical incorporation of the drug into a particulate colloidal system, for instance reverse micelles, noisome, micro- and nano-spheres, erythrocytes, and polymers and liposomes. Among these carriers, liposomes have been most studied. Their attractiveness lies in their composition, which makes them biodegradable and biocompatible. Liposome involves an aqueous core entrapped by one or more bilayers composed of natural or synthetic lipids. They are composed of natural phospholipids that are biologically inert and feebly immunogenic, and they have low inherent toxicity. Furthermore, drugs with different lipophilicities can be encapsulated into liposomes: strongly lipophilic drugs are entrapped almost totally in the lipid bilayer, intensely hydrophilic drugs are located entirely in the aqueous compartment, and drugs with intermediary logP effortlessly partition between the lipid and aqueous phases, both in the bilayer and in the aqueous core [15].

The present review will briefly explain the characteristics of liposomes and explore the related problems and solutions proposed, with a focus on liposome preparation, characterizations, affecting factors, advantages, and disadvantages. In particular, we return to the literature relating to high-stability, long-circulating liposomes (stealth liposomes), and their field of application.

### Classification of liposomes

The liposome size can vary from very small (0.025 μm) to large (2.5 μm) vesicles. Moreover, liposomes may have one or bilayer membranes. The vesicle size is an acute parameter in determining the circulation half-life of liposomes, and both size and number of bilayers affect the amount of drug encapsulation in the liposomes. On the basis of their size and number of bilayers, liposomes can also be classified into one of two categories: (1) multilamellar vesicles (MLV) and (2) unilamellar vesicles. Unilamellar vesicles can also be classified into two categories: (1) large unilamellar vesicles (LUV) and (2) small unilamellar vesicles (SUV) [16]. In unilamellar liposomes, the vesicle has a single phospholipid bilayer sphere enclosing the aqueous solution. In multilamellar liposomes, vesicles have an onion structure. Classically, several unilamellar vesicles will form on the inside of the other with smaller size, making a multilamellar structure of concentric phospholipid spheres separated by layers of water [17].

### Methods of liposome preparation

#### General methods of preparation

All the methods of preparing the liposomes involve four basic stages:

1. Drying down lipids from organic solvent.

2. Dispersing the lipid in aqueous media.

3. Purifying the resultant liposome.

4. Analyzing the final product.

#### Method of liposome preparation and drug loading

The following methods are used for the preparation of liposome:

1. Passive loading techniques

2. Active loading technique.

Passive loading techniques include three different methods:

1. Mechanical dispersion method.

2. Solvent dispersion method.

3. Detergent removal method (removal of non-encapsulated material) [18,19].

#### Mechanical dispersion method

The following are types of mechanical dispersion methods:

1.1. Sonication.

1.2. French pressure cell: extrusion.

1.3. Freeze-thawed liposomes.

1.4. Lipid film hydration by hand shaking, non-hand. shaking or freeze drying.

1.5. Micro-emulsification.

1.6. Membrane extrusion.

1.7. Dried reconstituted vesicles [18,19].

##### Sonication

Sonication is perhaps the most extensively used method for the preparation of SUV. Here, MLVs are sonicated either with a bath type sonicator or a probe sonicator under a passive atmosphere. The main disadvantages of this method are very low internal volume/encapsulation efficacy, possible degradation of phospholipids and compounds to be encapsulated, elimination of large molecules, metal pollution from probe tip, and presence of MLV along with SUV [18].

There are two sonication techniques:

a) Probe sonication. The tip of a sonicator is directly engrossed into the liposome dispersion. The energy input into lipid dispersion is very high in this method. The coupling of energy at the tip results in local hotness; therefore, the vessel must be engrossed into a water/ice bath. Throughout the sonication up to 1 h, more than 5% of the lipids can be de-esterified. Also, with the probe sonicator, titanium will slough off and pollute the solution.

b) Bath sonication. The liposome dispersion in a cylinder is placed into a bath sonicator. Controlling the temperature of the lipid dispersion is usually easier in this method, in contrast to sonication by dispersal directly using the tip. The material being sonicated can be protected in a sterile vessel, dissimilar the probe units, or under an inert atmosphere [20].

##### French pressure cell: extrusion

French pressure cell involves the extrusion of MLV through a small orifice [18]. An important feature of the French press vesicle method is that the proteins do not seem to be significantly pretentious during the procedure as they are in sonication [21]. An interesting comment is that French press vesicle appears to recall entrapped solutes significantly longer than SUVs do, produced by sonication or detergent removal [22-24].

The method involves gentle handling of unstable materials. The method has several advantages over sonication method [25]. The resulting liposomes are rather larger than sonicated SUVs. The drawbacks of the method are that the high temperature is difficult to attain, and the working volumes are comparatively small (about 50 mL as the maximum) [18,19].

##### Freeze-thawed liposomes

SUVs are rapidly frozen and thawed slowly. The short-lived sonication disperses aggregated materials to LUV. The creation of unilamellar vesicles is as a result of the fusion of SUV throughout the processes of freezing and thawing [26-28]. This type of synthesis is strongly inhibited by increasing the phospholipid concentration and by increasing the ionic strength of the medium. The encapsulation efficacies from 20% to 30% were obtained [26].

#### Solvent dispersion method

##### Ether injection (solvent vaporization)

A solution of lipids dissolved in diethyl ether or ether-methanol mixture is gradually injected to an aqueous solution of the material to be encapsulated at 55°C to 65°C or under reduced pressure. The consequent removal of ether under vacuum leads to the creation of liposomes. The main disadvantages of the technique are that the population is heterogeneous (70 to 200 nm) and the exposure of compounds to be encapsulated to organic solvents at high temperature [29,30].

##### Ethanol injection

A lipid solution of ethanol is rapidly injected to a huge excess of buffer. The MLVs are at once formed. The disadvantages of the method are that the population is heterogeneous (30 to 110 nm), liposomes are very dilute, the removal all ethanol is difficult because it forms into azeotrope with water, and the probability of the various biologically active macromolecules to inactivate in the presence of even low amounts of ethanol is high [31].

##### Reverse phase evaporation method

This method provided a progress in liposome technology, since it allowed for the first time the preparation of liposomes with a high aqueous space-to-lipid ratio and a capability to entrap a large percentage of the aqueous material presented. Reverse-phase evaporation is based on the creation of inverted micelles. These inverted micelles are shaped upon sonication of a mixture of a buffered aqueous phase, which contains the water-soluble molecules to be encapsulated into the liposomes and an organic phase in which the amphiphilic molecules are solubilized. The slow elimination of the organic solvent leads to the conversion of these inverted micelles into viscous state and gel form. At a critical point in this process, the gel state collapses, and some of the inverted micelles were disturbed. The excess of phospholipids in the environment donates to the formation of a complete bilayer around the residual micelles, which results in the creation of liposomes. Liposomes made by reverse phase evaporation method can be made from numerous lipid formulations and have aqueous volume-to-lipid ratios that are four times higher than hand-shaken liposomes or multilamellar liposomes [19,20].

Briefly, first, the water-in-oil emulsion is shaped by brief sonication of a two-phase system, containing phospholipids in organic solvent such as isopropyl ether or diethyl ether or a mixture of isopropyl ether and chloroform with aqueous buffer. The organic solvents are detached under reduced pressure, resulting in the creation of a viscous gel. The liposomes are shaped when residual solvent is detached during continued rotary evaporation under reduced pressure. With this method, high encapsulation efficiency up to 65% can be obtained in a medium of low ionic strength for example 0.01 M NaCl. The method has been used to encapsulate small, large, and macromolecules. The main drawback of the technique is the contact of the materials to be encapsulated to organic solvents and to brief periods of sonication. These conditions may possibly result in the breakage of DNA strands or the denaturation of some proteins [32]. Modified reverse phase evaporation method was presented by Handa et al., and the main benefit of the method is that the liposomes had high encapsulation efficiency (about 80%) [33].

#### Detergent removal method (removal of non-encapsulated material)

##### Dialysis

The detergents at their critical micelle concentrations (CMC) have been used to solubilize lipids. As the detergent is detached, the micelles become increasingly better-off in phospholipid and lastly combine to form LUVs. The detergents were removed by dialysis [34-36]. A commercial device called LipoPrep (Diachema AG, Switzerland), which is a version of dialysis system, is obtainable for the elimination of detergents. The dialysis can be performed in dialysis bags engrossed in large detergent free buffers (equilibrium dialysis) [17].

##### Detergent (cholate, alkyl glycoside, Triton X-100) removal of mixed micelles (absorption)

Detergent absorption is attained by shaking mixed micelle solution with beaded organic polystyrene adsorbers such as XAD-2 beads (SERVA Electrophoresis GmbH, Heidelberg, Germany) and Bio-beads SM2 (Bio-RadLaboratories, Inc., Hercules, USA). The great benefit of using detergent adsorbers is that they can eliminate detergents with a very low CMC, which are not entirely depleted.

##### Gel-permeation chromatography

In this method, the detergent is depleted by size special chromatography. Sephadex G-50, Sephadex G-l 00 (Sigma-Aldrich, MO, USA), Sepharose 2B-6B, and Sephacryl S200-S1000 (General Electric Company, Tehran, Iran) can be used for gel filtration. The liposomes do not penetrate into the pores of the beads packed in a column. They percolate through the inter-bead spaces. At slow flow rates, the separation of liposomes from detergent monomers is very good. The swollen polysaccharide beads adsorb substantial amounts of amphiphilic lipids; therefore, pre-treatment is necessary. The pre-treatment is done by pre-saturation of the gel filtration column by lipids using empty liposome suspensions.

#### Dilution

Upon dilution of aqueous mixed micellar solution of detergent and phospholipids with buffer, the micellar size and the polydispersity increase fundamentally, and as the system is diluted beyond the mixed micellar phase boundary, a spontaneous transition from polydispersed micelles to vesicles occurs.

### Stealth liposomes and conventional liposomes

Although liposomes are like biomembranes, they are still foreign objects of the body. Therefore, liposomes are known by the mononuclear phagocytic system (MPS) after contact with plasma proteins. Accordingly, liposomes are cleared from the blood stream.

These stability difficulties are solved through the use of synthetic phospholipids, particle coated with amphipathic polyethylene glycol, coating liposomes with chitin derivatives, freeze drying, polymerization, microencapsulation of gangliosides [17].

Coating liposomes with PEG reduces the percentage of uptake by macrophages and leads to a prolonged presence of liposomes in the circulation and, therefore, make available abundant time for these liposomes to leak from the circulation through leaky endothelium.

A stealth liposome is a sphere-shaped vesicle with a membrane composed of phospholipid bilayer used to deliver drugs or genetic material into a cell. A liposome can be composed of naturally derived phospholipids with mixed lipid chains coated or steadied by polymers of PEG and colloidal in nature. Stealth liposomes are attained and grown in new drug delivery and in controlled release. This stealth principle has been used to develop the successful doxorubicin-loaded liposome product that is presently marketed as Doxil (Janssen Biotech, Inc., Horsham, USA) or Caelyx (Schering-Plough Corporation, Kenilworth, USA) for the treatment of solid tumors. Recently impressive therapeutic improvements were described with the useof corticosteroid-loaded liposome in experimental arthritic models. The concerning on the application of stealth liposomes has been on their potential to escape from the blood circulation. However, long circulating liposome may also act as a reservoir for prolonged release of a therapeutic agent. Pharmacological action of vasopressin is formulated in long circulating liposome [37,38].

#### Drug loading in liposomes

Drug loading can be attained either passively (i.e., the drug is encapsulated during liposome formation) or actively (i.e., after liposome formation). Hydrophobic drugs, for example amphotericin B taxol or annamycin, can be directly combined into liposomes during vesicle formation, and the amount of uptake and retention is governed by drug-lipid interactions. Trapping effectiveness of 100% is often achievable, but this is dependent on the solubility of the drug in the liposome membrane. Passive encapsulation of water-soluble drugs depends on the ability of liposomes to trap aqueous buffer containing a dissolved drug during vesicle formation. Trapping effectiveness (generally <30%) is limited by the trapped volume delimited in the liposomes and drug solubility. On the other hand, water-soluble drugs that have protonizable amine functions can be actively entrapped by employing pH gradients [39], which can result in trapping effectiveness approaching 100% [40].

#### Freeze-protectant for liposomes (lyophilization)

Natural excerpts are usually degraded because of oxidation and other chemical reactions before they are delivered to the target site. Freeze-drying has been a standard practice employed to the production of many pharmaceutical products. The overwhelming majority of these products are lyophilized from simple aqueous solutions. Classically, water is the only solvent that must be detached from the solution using the freeze-drying process, but there are still many examples where pharmaceutical products are manufactured via a process that requires freeze-drying from organic co-solvent systems [14].

Freeze-drying (lyophilization) involves the removal of water from products in the frozen state at tremendously low pressures. The process is normally used to dry products that are thermo-labile and would be demolished by heat-drying. The technique has too much potential as a method to solve long-term stability difficulties with admiration to liposomal stability. Studies showed that leakage of entrapped materials may take place during the process of freeze-drying and on reconstitution. Newly, it was shown that liposomes when freeze-dried in the presence of adequate amounts of trehalose (a carbohydrate commonly found at high concentrations in organism) retained as much as 100% of their original substances. It shows that trehalose is an excellent cryoprotectant (freeze-protectant) for liposomes. Freeze-driers range in size from small laboratory models to large industrial units available from pharmaceutical equipment suppliers [41].

#### Mechanism of transportation through liposome

The limitations and benefits of liposome drug carriers lie critically on the interaction of liposomes with cells and their destiny *in vivo* after administration. *In vivo* and *in vitro* studies of the contacts with cells have shown that the main interaction of liposomes with cells is either simple adsorption (by specific interactions with cell-surface components, electrostatic forces, or by non-specific weak hydrophobic) or following endocytosis (by phagocytic cells of the reticuloendothelial system, for example macrophages and neutrophils).

Fusion with the plasma cell membrane by insertion of the lipid bilayer of the liposome into the plasma membrane, with simultaneous release of liposomal content into the cytoplasm, is much rare. The fourth possible interaction is the exchange of bilayer components, for instance cholesterol, lipids, and membrane-bound molecules with components of cell membranes. It is often difficult to determine what mechanism is functioning, and more than one may function at the same time [42-44].

#### Fusogenic liposomes and antibody-mediated liposomes in cancer therapy

It has been infrequently well-known that a powerful anticancer drug, especially one that targets the cytoplasm or cell nucleus, does not work due to the low permeability across a plasma membrane, degradation by lysosomal enzymes through an endocytosis-dependent pathway, and other reasons. Thus, much attention on the use of drug delivery systems is focused on overcoming these problems, ultimately leading to the induction of maximal ability of anti-cancer drug. In this respect, a new model for cancer therapy using a novel drug delivery system, fusogenic liposome [45], was developed.

Fusogenic liposomes are poised of the ultraviolet-inactivated Sendai virus and conventional liposomes. Fusogenic liposomes effectively and directly deliver their encapsulated contents into the cytoplasm using a fusion mechanism of the Sendai virus, whereas conventional liposomes are taken up by endocytosis by phagocytic cells of the reticuloendothelial system, for example macrophages and neutrophils. Thus, fusogenic liposome is a good candidate as a vehicle to deliver drugs into the cytoplasm in an endocytosis-independent manner [45].

Liposomal drug delivery systems provide steady formulation, provide better pharmacokinetics, and make a degree of ‘passive’ or ‘physiological’ targeting to tumor tissue available. However, these transporters do not directly target tumor cells. The design modifications that protect liposomes from unwanted interactions with plasma proteins and cell membranes which differed them with reactive carriers, for example cationic liposomes, also prevent interactions with tumor cells. As an alternative, after extravasation into tumor tissue, liposomes remain within tumor stroma as a drug-loaded depot. Liposomes ultimately become subject to enzymatic degradation and/or phagocytic attack, leading to release of drug for subsequent diffusion to tumor cells. The next generation of drug carriers under development features directs molecular targeting of cancer cells via antibody-mediated or other ligand-mediated interactions [17,45].

### Applications of liposomes in medicine and pharmacology

Applications of liposomes in medicine and pharmacology can be divided into diagnostic and therapeutic applications of liposomes containing various markers or drugs, and their use as a tool, a model, or reagent in the basic studies of cell interactions, recognition processes, and mode of action of certain substances [43].

Unfortunately, many drugs have a very narrow therapeutic window, meaning that the therapeutic concentration is not much lower than the toxic one. In several cases, the toxicity can be reduced or the efficacy can be enhanced by the use of a suitable drug carrier which alters the temporal and spatial delivery of the drug, i.e., its biodistribution and pharmacokinetics. It is clear from many pre-clinical and clinical studies that drugs, for instance antitumor drugs, parceled in liposome demonstration reduced toxicities, while retentive enhanced efficacy.

Advances in liposome design are leading to new applications for the delivery of new biotechnology products, for example antisense oligonucleotides, cloned genes, and recombinant proteins. A vast literature define the viability of formulating wide range of conservative drugs in liposomes, frequently resultant in improved therapeutic activity and/or reduced toxicity compared with the free drug. As a whole, changed pharmacokinetics for liposomal drugs can lead to improved drug bioavailability to particular target cells that live in the circulation, or more prominently, to extravascular disease sites, for example, tumors. Recent improvements include liposomal formulations of all-*trans*-retinoic acid [46,47] and daunorubicin [48-51], which has received Food and Drug Administration consent as a first-line treatment of AIDS-related advanced Kaposi's sarcoma. Distinguished examples are vincristine, doxorubicin, and amphotericin B [38].

The benefits of drug load in liposomes, which can be applied as (colloidal) solution, aerosol, or in (semi) solid forms, such as creams and gels, can be summarized into seven categories [44] (Table 2):

**Table 2:** Benefits of drug load in liposomes

**Benefits of drug load in liposome**	**Examples**
1. Improved solubility of lipophilic and amphiphilic drugs	Amphotericin B, porphyrins, minoxidil, some peptides, and anthracyclines, respectively; hydrophilic drugs, such as anticancer agent doxorubicin or acyclovir
2. Passive targeting to the cells of the immune system, especially cells of the mononuclear phagocytic system	Antimonials, amphotericin B, porphyrins, vaccines, immunomodulators
3. Sustained release system of systemically or locally administered liposomes	Doxorubicin, cytosine arabinoside, cortisones, biological proteins or peptides such as vasopressin
4. Site-avoidance mechanism	Doxorubicin andamphotericin B
5. Site-specific targeting	Anti-inflammatory drugs, anti-cancer, anti-infection
6. Improved transfer of hydrophilic, charged molecules	Antibiotics, chelators, plasmids, and genes
7. Improved penetration into tissues	Corticosteroids, anesthetics, and insulin

#### Liposomes in parasitic diseases and infections

From the time when conventional liposomes are digested by phagocytic cells in the body after intravenous management, they are ideal vehicles for the targeting drug molecules into these macrophages. The best known instances of this ‘Trojan horse-like’ mechanism are several parasitic diseases which normally exist in the cell of MPS. They comprise leishmaniasis and several fungal infections.

Leishmaniasis is a parasitic infection of macrophages which affects over 100 million people in tropical regions and is often deadly. The effectual dose of drugs, mostly different antimonials, is not much lower than the toxic one. Liposomes accumulate in the very same cell population which is infected, and so an ideal drug delivery vehicle was proposed [52]. Certainly, the therapeutic index was increased in rodents as much as several hundred times upon administration of the drug in various liposomes. Unexpectedly, and unfortunately, there was not much interest to scale up the formulations and clinically approve them after several very encouraging studies dating back to 1978. Only now, there are several continuing studies with various anti-parasitic liposome formulations in humans. These formulations use mostly ionosphere amphotericin B and are transplanted from very successful and prolific area of liposome formulations in antifungal therapy.

The best results reported so far in human therapy are probably liposomes as carriers foramphotericin B in antifungal therapies. This is the drug of choice in dispersed fungal infections which often in parallel work together with chemotherapy, immune system, or AIDS, and is frequently fatal. Unfortunately, the drug itself is very toxic and its dosage is limited due to its ionosphere and neurotoxicity. These toxicities are normally related with the size of the drug molecule or its complex. Obviously, liposome encapsulation inhibits the accumulation of drug in these organs and radically reduces toxicity [53]. Furthermore, often, the fungus exists in the cells of the mononuclear phagocytic system; therefore, the encapsulation results in reduced toxicity and passive targeting. These benefits, however, can be associated with any colloidal drug carrier. Certainly, similar improvements in therapy were observed with stable mixed micellar formulations and micro-emulsions [54]. Additionally, it seems that many of the early liposomal preparations were in actual fact liquid crystalline colloidal particles rather than self-closed MLV. Since the lives of the first terminally ill patients (who did not rely to all the conventional therapies) were saved [53], many patients were very effectively treated with diverse of amphotericin B formulations.

Comparable methods can be achieved in antiviral and antibacterial therapies [55]. Most of the antibiotics, however, are orally available; liposome encapsulation can be considered only in the case of very potent and toxic ones which are administered parenterally. The preparation of antibiotic-loaded liposomes at sensibly high drug-to-lipid ratios may not be easy because of the interactions of these molecules with bilayers and high densities of their aqueous solutions which often force liposomes to float as a creamy layer on the top of the tube. Several other ways, for instance, topical or pulmonary (by inhalation) administration are being considered also. Liposome-encapsulated antivirals (for example ribavirin, azidothymidine, or acyclovir) have also shown to reduce toxicity; currently, more detailed experiments are being performed in relation to their efficacy.

#### Liposomes in anticancer therapy

Numerous different liposome formulations of numerous anticancer agents were shown to be less toxic than the free drug [56-59]. Anthracyclines are drugs which stop the growth of dividing cells by intercalating into the DNA and, thus, kill mainly rapidly dividing cells. These cells are not only in tumors but are also in hair, gastrointestinal mucosa, and blood cells; therefore, this class of drug is very toxic. The most used and studied is Adriamycin (commercial name for doxorubicin HCl; Ben Venue Laboratories, Bedford, Ohio). In addition to the above-mentioned acute toxicities, its dosage is limited by its increasing cardio toxicity. Numerous diverse formulations were tried. In most cases, the toxicity was reduced to about 50%. These include both acute and chronic toxicities because liposome encapsulation reduces the delivery of the drug molecules towards those tissues. For the same reason, the efficiency was in many cases compromised due to the reduced bioavailability of the drug, especially if the tumor was not phagocytic or located in the organs of mononuclear phagocytic system. In some cases, such as systemic lymphoma, the effect of liposome encapsulation showed enhanced efficacy due to the continued release effect, i.e., longer presence of therapeutic concentrations in the circulation [60-62], while in several other cases, the sequestration of the drug into tissues of mononuclear phagocytic system actually reduced its efficacy.

Applications in man showed, in general, reduced toxicity and better tolerability of administration with not too encouraging efficacy. Several different formulations are in different phases of clinical studies and show mixed results.

## Conclusions

Liposomes have been used in a broad range of pharmaceutical applications. Liposomes are showing particular promise as intracellular delivery systems for anti-sense molecules, ribosomes, proteins/peptides, and DNA. Liposomes with enhanced drug delivery to disease locations, by ability of long circulation residence times, are now achieving clinical acceptance. Also, liposomes promote targeting of particular diseased cells within the disease site. Finally, liposomal drugs exhibit reduced toxicities and retain enhanced efficacy compared with free complements. Only time will tell which of the above applications and speculations will prove to be successful. However, based on the pharmaceutical applications and available products, we can say that liposomes have definitely established their position in modern delivery systems.

## Competing interests

The authors declare that they have no competing interests.

## Authors’ contributions

SWJ conceived the study and participated in its design and coordination. NZ participated in the sequence alignment and drafted the manuscript. AA, RRS, SD, YH, MS, MK, and KNK helped in drafting the manuscript. All authors read and approved the final manuscript.
